# Supplementary figures and images for: Diversity and conservation of mammals in indigenous territories of southern Mexico: proposal for an “Archipelago Reserve”
Source: PeerJ. 2023 Nov 7;11:e16345. doi: 10.7717/peerj.16345 (PMC10637252; doi:10.7717/peerj.16345)

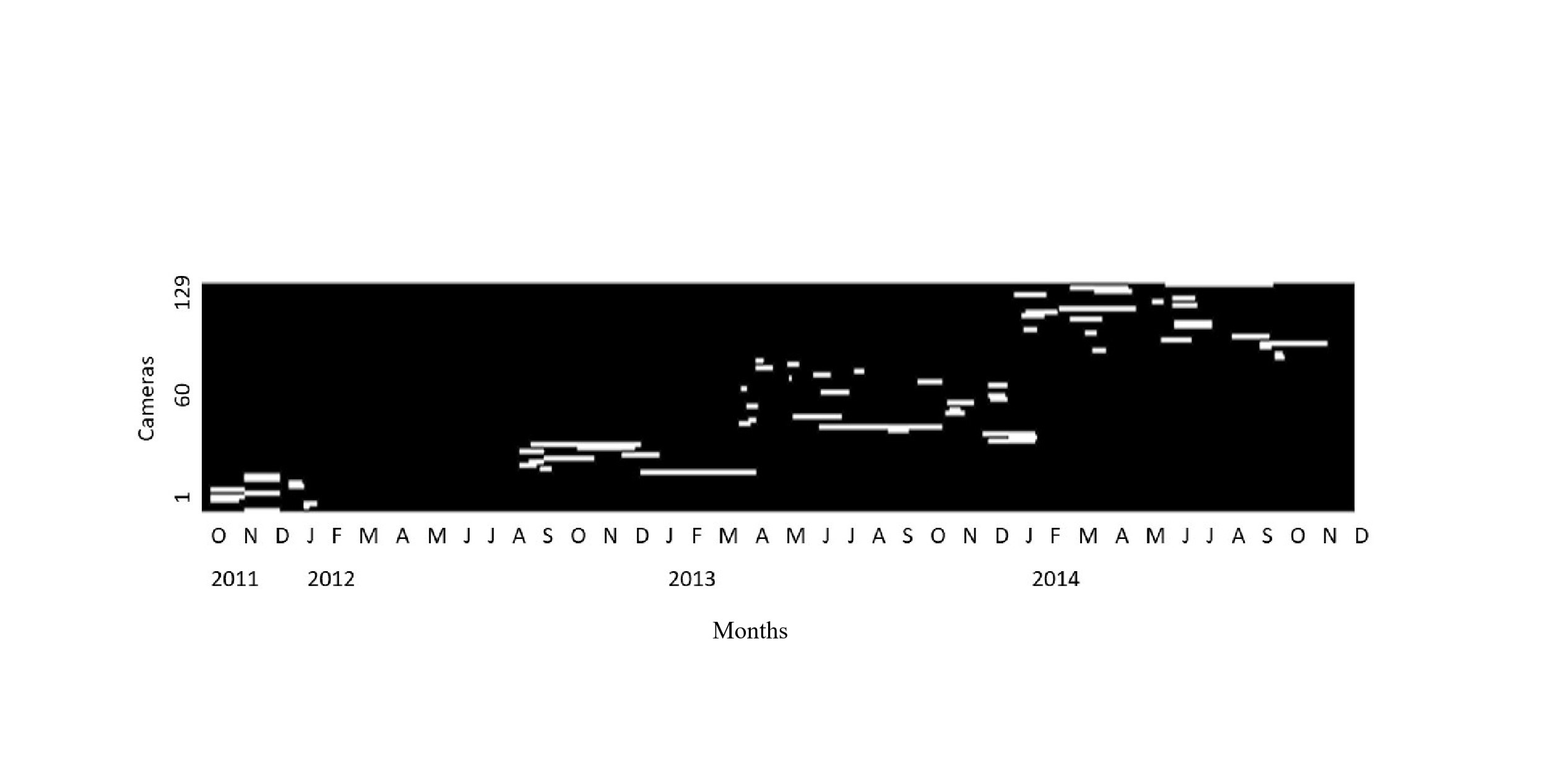

Supplement: Supplemental Information 2 [file peerj-11-16345-s002.jpg]
